# Evidence of Zika virus circulation in human and livestock in Chad

**DOI:** 10.1016/j.virusres.2024.199492

**Published:** 2024-11-20

**Authors:** François Chable de la Héronnière, Jonathan Barthelemy, Guy R Takoudjou Dzomo, Fatima Abdelrazakh, Oumaima Djarma, Lucas Auguste, Abderrazzack A Fouda, Chatté Adawaye, Laurent Andreoletti, Mahamat Fayiz Abakar, Yannick Simonin, Sara Salinas, Franck JD Mennechet

**Affiliations:** a Pathogenesis and Control of Chronic and Emerging Infections (PCCEI), INSERM U1058, University of Montpellier, French Blood Establishment (EFS), Montpellier, France; b University Hospital Complex "Le Bon Samaritain", N'Djamena, Chad; c Livestock Research Institute for Development (IRED), N'Djamena, Chad; d Ministry of Public Health and Solidarity, N'Djamena, Chad; e Toumaï University, N'Djamena, Chad; f Faculty of Human Health Sciences, N'Djamena, Chad; g Virology laboratory, Faculty of Medicine, INSERM U1320, University of Reims Champagne Ardennes, and UHC of Reims, France; h Academic Hospital of Reims, Robert Debré, Virology Department, Reims, France

**Keywords:** Zika virus, seroneutralization test, Chad, seroprevalence

## Abstract

Zika virus (ZIKV) is a major public health problem worldwide. After several reported outbreaks, the current extent of infections caused by this orthoflavivirus in the Sahel remains to be explored. We investigated the prevalence of neutralizing antibodies against ZIKV in the general population, in HIV-infected individuals and in livestock in Chad using a seroneutralization assay that ensures high specificity level. In this retrospective serological serosurveillance investigation, we estimated the neutralizing seroprevalence to be approximately 26 % (18/69) in healthy Chadian participants and 17 % (16/94) in HIV-infected individuals, with no statistical difference between these two subgroups or genders. In addition, we found an overall ZIKV prevalence of 14 % (8/59) in small ruminants (sheep and goats) living in the Lake Chad Basin area, demonstrating virus circulation in animals. Our pilot study shows for the first-time evidence of ZIKV circulation in humans and in livestock in Chad in close interaction with humans, and highlights the main challenges associated with this virus in Sahelian areas.

## Highlights

• First evidence of Zika virus circulation in Chad using a seroneutralization test.
• HIV status did not affect Zika virus seroprevalence rate or average NAb titers.
• Zika virus-specific NAb were also found in the livestock basin in Chad.
• Zika virus circulation is likely underestimated in Chad.
• Livestock testing could help monitor ZIKV in Chad.

## 1 Introduction

Zika virus (ZIKV) is a single-stranded positive RNA virus that belong to the *Flaviviridae* family and the *Orthoflavivirus* genus. It is an arbovirus (arthropod-borne virus) that is typically transmitted horizontally by hematophagous arthropods from one vertebrate host to another and can cause a variety of human diseases with different symptoms and epidemiologic characteristics. Recent decades have been characterized by dramatic epidemics of emerging and re-emerging arboviruses, particularly in Africa, which have led to further serious threats to public health on the continent (Pierson and Diamond, 2020). However, information on outbreaks of arboviruses in Africa is limited and likely to be largely unreported, which, combined with a high mutation rate, raises the possibility of new epidemic strains emerging in the coming years. ZIKV is one of the major emerging orthoflaviviruses, isolated for the first time in 1947 in the Zika Forest in Uganda (Musso and Gubler, 2016) from a sentinel Rhesus monkey. The virus is usually transmitted by *Aedes (aegypti* and *albopictus)* mosquitoes species (Agboli et al., 2021). ZIKV can also be transmitted vertically from mother to fetus, as well as through sexual intercourse, blood or blood products transfusions, or organ transplants (WHO, 2022). ZIKV infection can cause adverse congenital outcomes (CZS) such as congenital brain abnormalities in fetuses but also Guillain-Barre syndrome in adults (Liang et al., 2019). However, most people (∼60 %) infected with ZIKV do not present symptomatic forms, usually manifesting as a self-limited febrile episode, which adds to the factors that could cause a ZIKV epidemic to go unnoticed in Africa (Nutt and Adams, 2017). ZIKV circulates as two genetic lineages, “African” and “Asian”, which differ in virulence and epidemiological properties. The Asian lineage being apparently more associated with CZS, but is also the most studied lineage, as it was responsible for outbreaks in the Americas (Simonin et al., 2017).

Because ZIKV is found in all body fluids (Calvet et al., 2023), it may also be transmitted through sexual contact and blood transfusions in significant and possibly underestimated proportions (Allard et al., 2017). Because the spread of vector-borne viruses like ZIKV in tropical and subtropical regions has a complicated impact on the immunopathogenesis of other endemic viruses like HIV, interactions between ZIKV infection and HIV infection is therefore receiving particular attention (Rakotomalala et al., 2023; Rothan et al., 2018).

HIV prevalence is much higher in sub-Saharan Africa than in most other parts of the world (IBRD, IDA, 2023). Nevertheless, relative uncertainties remain as to the exact HIV prevalence levels in a significant number of areas of sub-Saharan zones (Dwyer-Lindgren et al., 2019). In Chad, the national HIV prevalence rate is estimated at 1 % in 2022 according to UNAIDS among adult, compared to a global average of ∼0.7 % (UNAIDS, 2022).

In addition to ZIKV, the most widespread orthoflaviviruses of concern in Sahelian Africa are dengue virus (DENV), yellow fever virus (YFV), West Nile virus (WNV) and the Japanese encephalitis virus (JEV) (Pierson and Diamond, 2020). Regarding arboviruses in Chad, YFV has been endemic for a long time (Djarma et al., 2021). According to the Chadian government, 2550 suspected cases of YFV were recorded between 2021 and 2023, including 74 deaths (Govt Chad and WHO, 2022). Epidemics of the DENV and chikungunya virus (CHIKV), an alphavirus, were first reported by the World Health Organization (WHO) in 2020 and 2023, respectively (WHO, 2020a, 2023a). In November 2023, 1589 suspected cases of DENV were reported in twelve health districts in four provinces of Chad, including 63 confirmed cases and one death (Africa CDC, 2023). Between July and September 2020, a total of 38,140 cases of CHIKV were notified, mainly in the Abéché and Biltine health districts (WHO, 2020b). The clinical diagnosis for both viruses was confirmed by the Yaoundé Pasteur laboratory. Although circulation of ZIKV is suspected in Chad, it has not yet been demonstrated. Unfortunately, very little additional information is available in Chad concerning ZIKV or arboviroses in general, mainly due to the lack of identification and diagnostic tools in Central Africa. In Chad's neighboring countries, the presence of ZIKV has nevertheless been reported by various seroprevalence methods and on heterogeneous populations in Cameroon, Niger, Nigeria and Sudan.

In this work, we present data obtained in a pilot surveillance study on ZIKV neutralizing seroprevalence in the general population of the city of N'Djamena, Chad, as well as in people living with HIV (PLHIV). From a one-health perspective, we also assessed ZIKV seroprevalence in livestock (sheep and goats) in the area of the Lake Chad Basin, providing to our knowledge first epidemiological evidence of the virus circulation in Chad among both human beings and animals.

## 2 Methods

### 2.1 Samples characteristics and viruses

**Fig. 1:**
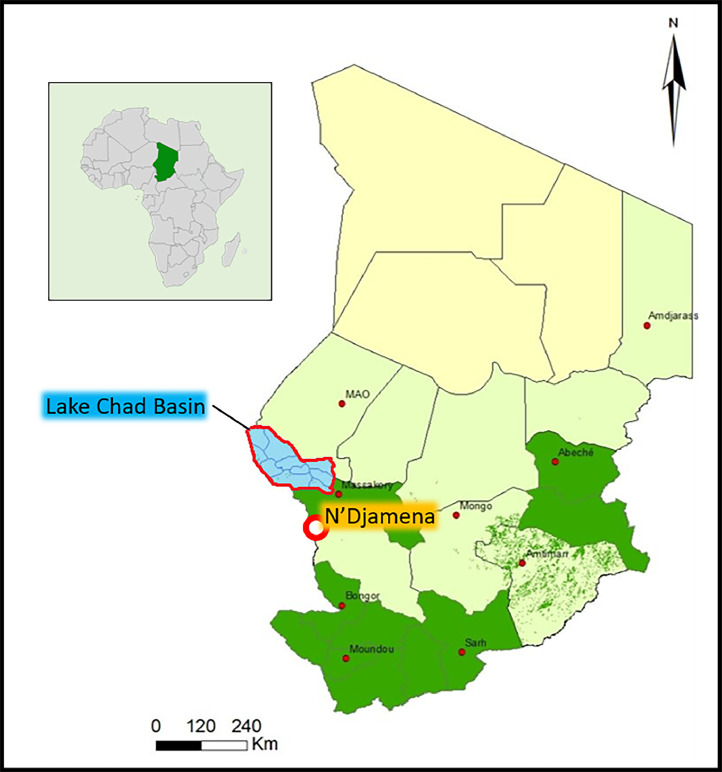
Sampling location: Human plasma samples were collected in the city of N'Djamena, the capital of Chad, and veterinary samples (sheep and goats) were collected from cattle in the Lake Chad Basin region.

The study involved a combined total of 163 human plasma samples, taken from the general population (69/163 – 42.3 %) and PLHIV (94/163 – 57.6 %) from the city of N'Djamena, Chad (Fig. 1). Human samples were collected for previous and ongoing studies and reused for the present study. Human samples were collected in 2017 and 2021 at Hôpital du Bon Samaritain (CHU-BS) (HIV- / *n* = 69) from healthy volunteer donors who came for routine check-ups or during medical follow-up of HIV-positive patients (PLHIV (HIV+ / *n* = 94)) at the Programme Sectoriel de Lutte contre le SIDA (PSLS), in the city of N'Djamena, Chad**.** Veterinary samples used in this study were provided from the “Institut de Recherche sur l'Elevage pour le Developpement” (IRED) of N'Djamena and were collected in 2020 among livestock (*n* = 59) (30 goats, 29 sheep) in the agricultural zone of the Lake Chad Basin (Fig. 1). The virus strains used for viral serum neutralization tests were the ArB41644 ZIKV strain of African lineage and the Africa 2 (Rhône 2705/France/2015-KX601692) USUV strain supplied by French Agency for Food, Environmental and Occupational Health and Safety (ANSES).

### 2.2 Ethical considerations

This study was carried out according to the principles of the declaration of Helsinki. Management approval for each study site was sought prior to the start of the study. All samples used for this study include written and signed informed consent from all participants, or from parents in the case of samples from participants who are under 18. Data have been rendered anonymous for publication purposes. Animal sampling from sheep and goats was carried out in accordance with national and institutional guidelines, as well as European Parliament Directive 2010/63/EU on animal experimentation. Directors of the CHU-BS and coordinator of the PSLS granted written permission to use the sample collection from HIV- participants, and HIV+ participants, respectively (N°0213/PT/PMT/MSPP/SE/SG/PSLSH_IST/23). The project has been approved by Chad's National Bioethics Committee (N°043-PT/PM/MESRI/SE/SG/CNBT/SG/2023) which indicated that samples could be reused for serological testing in biomedical research.

### 2.3 Enzyme-linked immunosorbent assay (ELISA)

For initial screening of samples for antibodies directed to a broad spectrum of orthoflaviviruses, a competitive ELISA kit for the detection of anti-pr-E (pan-orthoflavivirus) antibodies in multiple species was used according to the manufacturer instructions (Innovative Diagnostics Screen® ELISA ID Screen® Flavivirus Competition—France).

### 2.4 Cell culture and viral seroneutralization assays

Virus seroneutralization assays for ZIKV were performed on Vero cells in 96-well plates as previously described (Tinto et al., 2022a). Briefly, Vero cells were grown in complete DMEM medium containing 10 % heat-inactivated fetal calf serum (FCS) + antibiotics (AB) (1 % penicillin/streptomycin). Plasma samples were diluted in a cascade at a rate of one-third from 1/5 to 1/3645 in DMEM + 2 % FCS + AB and incubated 90 min at 37 °C + 5 % CO_2_ with the virus at 100 Tissue Culture Infectious Dose 50 per well (100-TCID50/well). Vero cells were then immediately added at 2.10^3^ per well and cytopathic effect observation performed 5 to 7 days post incubation based on controls conditions. Specific neutralizing antibodies (NAb) titers were calculated by determining the reciprocal of the last dilution at which no cytopathic effect was observed (Tinto et al., 2022b). To avoid false positives due to non-specific cell survival caused by high plasma concentrations, we set the positivity threshold at a 15-fold dilution (Relative NAb titer = 15) for both ZIKV and USUV. Plasma from patients vaccinated with YFV or infected with DENV were used as negative controls. In addition, all ZIKV serum-neutralization-positive samples tested negative in USUV serum-neutralization test, under similar experimental conditions.

### 2.5 Statistical analysis

Statistical analyses were performed using non-parametric Mann-Whitney tests to compare the level of NAb titers between groups. A p-value of <0.05 was considered statistically significant.

## 3 Results

### 3.1 ZIKV seroprevalence in Chad

The socio-demographic characteristics of the participants in this study are shown in the table 1.

**Table 1:** Socio-demographic characteristics of the participants in this study.

**Group ID**	**Overall**	**HIV -**	**HIV+**
**Number of sample (%)**	163 (100)	69 (42.3)	94 (57.6)
**Sex, n (%)**			
**Male**	71 (43.6)	33 (47.8)	38 (40.4)
**Female**	92 (56.4)	36 (52.2)	56 (59.6)
**Age range**	6 months – 69 years	6 months – 63 years	7 years – 69 years
**Mean age**	33.7 years	28.3 years	37.7 years

The samples collected included both adults and children, with 43.6 % being male and 56.4 % female, with an average age of 33.7 years (6 months to 69 years). Plasma samples were first analyzed by ELISA for the presence of anti-Pr-E (pan-orthoflavivirus) IgG and IgM. Of all samples tested, 93.2 % (152/163) were positive and three were considered doubtful. Positivity indicates the presence of specific antibodies against at least one orthoflavivirus, regardless of species. Positive and suspect samples were then tested for ZIKV-specific NAb by virus neutralization assay.

**Fig. 2:**
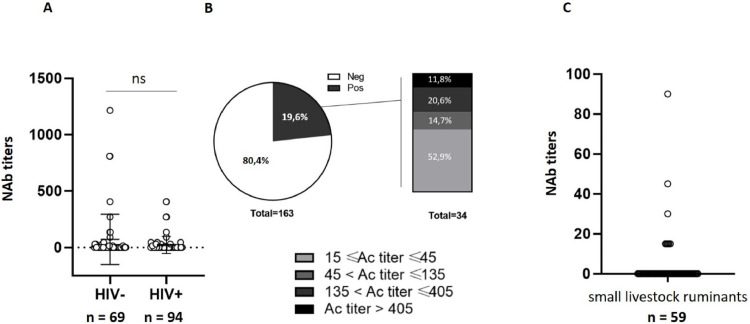
NAb seroprevalence against ZIKV in Chad among: (A) People living with HIV (HIV+) in comparison to control healthy participants (bars: mean ± SEM). (B) Percentage of positivity (NAb titer ≥ 15) among all samples (HIV+ and HIV-) (left part) and distribution (percentage) of NAb titers among positive samples (right part). NAb titers were arbitrarily stratified into the following categories: Neg: < 15 & Pos: (15–45), (46–135), (135–405), (> 406). (C) NAb seroprevalence against ZIKV among small ruminants (ovine/caprine) from the Lake Chad Basin livestock. ns: non-significant.

We found an overall NAb seroprevalence close to 20 % (32/163), with no significant difference between participants from the general population (26 % – 18/69) and those living with HIV (17 % – 16/94) (Fig. 2**A**), or in terms of seropositivity rate or average NAb titers among positive samples. We also found no significant difference in seroprevalence rate between samples taken in 2017 and those taken in 2021, or by gender **(not shown)**. Of the positive samples, approximately 50 % had relatively high NAb titer levels (> 45) (Fig. 2**B)**. Samples found doubtful for the anti-Pr-E ELISA assay were negative for anti-ZIKV NAb. Our studies also revealed the presence of specific NAb against ZIKV in approximately 13.5 % (8/59) of the samples collected in 2020 from small ruminants (sheep and goats) from the livestock areas of the Lake Chad basin (Fig. 2**C**).

## 4 Discussion

Herein, we serologically evidenced ZIKV circulation both in human beings and in livestock using seroneutralization assay. Although ZIKV had been previously detected in most neighboring countries, its presence in Chad had not yet been documented. In the present study, a first ELISA assay for anti-Pr-E (pan-orthoflavivirus) IgG and IgM revealed that >90 % of our samples were positive, indicating that most of our participants were immune to at least one orthoflavivirus and/or had been vaccinated against YFV. According to the WHO, the last estimate of YFV coverage in Chad was ∼45 % (WHO, 2023b), indicating nevertheless that a large proportion of the participants in our study showed an anti-pan-orthoflavivirus humoral response. These values are comparable to those obtained by our team on samples from Burkina Faso, which revealed a positivity rate of nearly 80 % among blood donor samples from 2020 (Tinto et al., 2022a). Due to the antigenic cross-reactivity between orthoflaviviruses, we then performed a virus neutralization assay to specifically assess and quantify ZIKV NAbs. To increase the robustness of our assays, we tested several control samples, such as plasma from patients vaccinated against YVF or who had contracted DENV fever. In addition, samples identified with a high level ZIKV-NAbs proved negative for USUV neutralization under similar experimental conditions. Using these controls, we selected a cut-off at a 15-fold dilution (Relative NAb titer = 15) for virus neutralization positivity. We estimated the overall NAb ZIKV seroprevalence rates at ∼20 % **(**Fig. 2**A and B)**, similar to what has been observed in Nigeria (∼28 %) (Kolawole et al., 2020; Mathé et al., 2018; Oluwole et al., 2022; Shaibu et al., 2021) and Niger (∼18 %) (Brès, 1970) but higher than in Cameroon (∼5 %) (Gake et al., 2017) and much lower to Sudan (∼63 %) (Soghaier et al., 2018), its neighboring countries. Although no seroprevalence studies have been carried out in the Central African Republic, the presence of the virus has been virologically documented in humans and in the transmission vector (Kamgang et al., 2020). Surprisingly, by comparison with the city of Maroua in Cameroon (2 %), we find a much higher seroprevalence rate, even though the two cities are <250 km apart. This could be explained by several factors that remain to be determined, such as differences in population density, a climate more or less conducive to vector transmission, or the presence of varying stagnant water spots between these cities as well as significant differences between prospective or retrospective epidemiological surveys and related workflows. In accord with others (Choyrum et al., 2022), we also did not observe significant differences on ZIKV seroprevalence rate between PLHIV and a control group (Fig. 2**A)**. By contrast, a lower ZIKV seroprevalence was observed in Nigeria for PLHIV by comparison with a control group (Mac et al., 2023), emphasizing the need for more larger serological investigations in HIV-1 infected patients living in various Sahelian areas using seroneutralization methods.

Our work and that of other teams, clearly shows that ZIKV circulation was detected in human beings, animals and transmission vectors in almost all the most populated areas of the Sahel. At the same time, we underline the many gaps in our knowledge about the extent of ZIKV-associated disease in the Sahel zone. Comparing our data with studies conducted in neighboring countries poses challenges due to the scarcity of studies, which are often outdated and focused on specific demographics like pregnant women or patients hospitalized with unknown fever. For Niger, the sole study available dates back over half a century (Brès, 1970). In Sudan, the eastern zone was exclusively explored, excluding the Darfur region, presumably because of the insecurity that has prevailed there for over 20 years. In Nigeria, studies have focused mainly on pregnant women, with very heterogeneous findings. As a result, we believe there is a need for greater standardization of the method used to assess ZIKV seroprevalence. Establishing effective ways to diagnose orthoflaviviruses has also been a long-standing global challenge. However, reaching a consensus on the methodology for conducting epidemiological surveys will undoubtedly encounter difficulties inherent to the Sahel, such as implementing neutralization tests, more suitable given the antigenic cross-reactivity of orthoflaviviruses, but requiring cell cultures. Nonetheless, we highlight here the major limitations of the implementation process of international recommendations for orthoflaviviruses, which do not sufficiently consider the particularities of sub-Saharan Africa. The challenges encountered in the management and control of orthoflavivirus such as ZIKV in Africa are also attributed to factors that favor the spread of vectors and increase the epidemiological potential. One hypothesis raised is that vertebrates might play a role of reservoir in the persistence of orthoflavivirus in the nature (Kuno et al., 2017), although more in-depth studies are needed to assess this possibility. Many orthoflavivirus-related epidemics go unnoticed in Africa in part because of the lack of understanding of the host-vector-orthoflavivirus interactions. Notable in this work, we revealed the presence of specific NAb against ZIKV in approximately 13.5 % of the samples collected from sheep and goats from the livestock areas of the Lake Chad Basin (Fig. 2**C)**, demonstrating, that ZIKV is circulating in this breeding ponds. Our observations are not sufficient to indicate that ZIKV circulates between livestock and humans, but they do suggest that livestock testing could be an interesting prospect for ZIKV surveillance in Chad. Further work will be needed to establish that these animals are indeed important in maintaining this virus in sylvatic foci and to determine whether ZIKV circulate between livestock and humans or not.

Moreover, the seroprevalence rate that we have observed in livestock is relatively high. By way of comparison, in the sylvatic cycle, non-human primates (NHPs) can be infected with ZIKV and could act as a reservoir for virus transmission, although this remains unclear. In a systematic review and meta-analysis of ZIKV seroprevalence in NHPs worldwide, the average seropositivity was 6 %, but reached 16 % in African NHPs (Mongkol et al., 2022).This study concurs with our own in terms of ZIKV seroprevalence in animals. Moreover, the Lake Chad Basin region is one of the most affected by malaria, suggesting a high prevalence of transmission vectors. This relatively high prevalence of ZIKV in the livestock in our study could be thus associated with the geographical location of the animals. Although we found a relatively high ZIKV seroprevalence in livestock, their relative NAb titers are low compared to humans. There is no clear explanation for these differences, as there are very few literatures investigating this question. One hypothesis is the substantial difference in immune response between these animals and humans. More in-depth studies of ZIKV seroprevalence in human populations in the Lake Chad Basin, as well as in livestock breeders, would provide a clearer picture of these questions.

In addition to the many challenges listed above, the orthoflavivirus situation in the Sahel is clearly camouflaged by the endemic reality of a high malaria prevalence. Numerous studies have indicated that orthoflavivirus infections have been mistaken for malaria (Kolawole et al., 2018). Indeed, countless other febrile illnesses, including bacterial, viral, and fungal infections, have a similar symptomatology to malaria and are regularly misdiagnosed as such (Nasir et al., 2017). Our study evidences the miss-detected circulation of the ZIKV in Chad. However, our pilot study has limitations. First, our sample size does not allow for statistical correlations between ZIKV seroprevalence and relevant sociodemographic factors. In addition, our study included only participants from urban areas in the city of N'Djamena, whereas most Chadians live in rural areas. Furthermore, although highly specific, seroneutralization assay provides no information on the genetic characteristics of the ZIKV strains involved in Chad. Therefore, our results are not only unrepresentative of the national population but are likely underestimating the overall ZIKV epidemiological picture in this country. We also studied samples collected during three periods only, 2017, 2020 for animals, and 2021, not allowing an accurate temporal analysis of the circulation of the virus in recent years. Including analysis methods that discriminate IgM from IgG would also provide information on the frequency of recent orthoflavivirus infections, as our ELISA assays do not discriminate between IgG and IgM.

## 5 Conclusion

In summary, we evidence the presence of detected NAb directed against ZIKV in around 1/5 of the participants including people with various HIV serological status, but also in the livestock around Lake Chad Basin. This pilot study clearly indicates the need for more in-depth and larger-scale studies to assess the extent of ZIKV infections in Chad. Furthermore, it underscores the necessity for better implementation of orthoflavivirus diagnosis and monitoring methods in Central Africa, incorporating a one-health approach.

## CRediT authorship contribution statement

**François Chable de la Héronnière:** Writing – original draft, Methodology, Investigation, Conceptualization. **Jonathan Barthelemy:** Formal analysis, Conceptualization. **Guy R Takoudjou Dzomo:** Writing – review & editing, Data curation. **Fatima Abdelrazakh:** Data curation. **Oumaima Djarma:** Data curation. **Lucas Auguste:** Formal analysis. **Abderrazzack A Fouda:** Data curation. **Chatté Adawaye:** Data curation. **Laurent Andreoletti:** Writing – review & editing, Formal analysis. **Mahamat Fayiz Abakar:** Investigation, Data curation. **Yannick Simonin:** Writing – review & editing, Methodology, Formal analysis, Conceptualization. **Sara Salinas:** Writing – review & editing, Methodology, Formal analysis, Conceptualization. **Franck JD Mennechet:** Writing – review & editing, Writing – original draft, Visualization, Validation, Supervision, Resources, Project administration, Investigation, Funding acquisition, Formal analysis, Data curation, Conceptualization.

## Declaration of competing interest

The authors declare that they have no known competing financial interests or personal relationships that could have appeared to influence the work reported in this paper.

## Data Availability

Data will be made available on request.
